# Trichophytobezoar Without Gastrointestinal Complaints

**DOI:** 10.1155/DTE.2.175

**Published:** 1996

**Authors:** G. M. Malik, M. Mubarik, A. Nisar, S. A. Kadla, M. S. Malik, M. D. Khan, B. A. Lone

**Affiliations:** 1 Government Medical College Srinagar Kashmir India; 2 F.A.C.G. R/O Kamerwari SK Coloney, IA Srinagar Kashmir 190 009 India

## Abstract

Gastrointestinal complaints may be the presenting symptoms of trichophytobezoar. We report a patient, who had an epigastric lump and enlarged lymph nodes on the left side of the neck, but without any gastrointestinal complaint. The clinical diagnosis suggested lymphoma or gastric cancer with secondary masses in the cervical lymph nodes. The diagnosis ultimately proved to be tubercular lymphadenitis on biopsy and gastric trichophytobezoar on endoscopy and laparotomy.


*Diagnostic and Therapeutic Endoscopy*, 1996, Vol. 2, pp. 175–177
Reprints available directly from the publisher
Photocopying permitted by license only

© 1996 OPA (Overseas Publishers Association)
Amsterdam B.V. Published in The Netherlands
by Harwood Academic Publishers GmbH
Printed in Singapore

### Trichophytobezoar Without Gastrointestinal Complaints

G. M. MALIK, M. MUBARIK, A. NISAR, S. A. KADLA, M. S. MALIK, M. D. KHAN and B. A. LONE

*Government Medical College, SrinagarKashmir, India*

*(Received November 27, 1994; in final form June 20, 1995)*

Gastrointestinal complaints may be the presenting symptoms of trichophytobezoar. We report a patient, who had an epigastric lump and enlarged lymph nodes on the left side of the neck, but without any gastrointestinal complaint. The clinical diagnosis suggested lymphoma or gastric cancer with secondary masses in the cervical lymph nodes. The diagnosis ultimately proved to be tubercular lymphadenitis on biopsy and gastric trichophytobezoar on endoscopy and laparotomy.

**KEY WORDS:** Lymphoma, carcinoma, tubercular, trichophytobezoar, endoscopy

### INTRODUCTION

Gastrointestinal bezoars result from ingestion of undigestible organic material such as hair (trichobezoar), vegetable and fruit fibers (phytobezoar), or a combination of both (trichophytobezoar). Bezoar form mainly in the stomach, with 90% occurring in women. Bezoars produce symptoms ranging from epigastric pain, nausea, vomiting, and obstruction to a mobile abdominal mass (1–3). We report on a case of gastric trichophytobezoar coincidentally associated with left cervical tubercular lymphadenopathy.

### CASE REPORT

A 42-year-old woman complained of an epigastrial mass. There was no history suggestive of any gastrointestinal disease. She did not complain of weight loss, fever, or night sweats. On general physical examination, the patient was well-built with mild pallor. She had left side cervical lymphadenopathy, which was nontender, smooth, and firm in consistency. Chest and cardiovascular examination results were normal. Abdominal examination revealed a scar of previous lower segment cesarian section, rectal divarication and an umbilical hernia. She had a 10 by 8 cm intraabdominal hard, nontender, nonpulsatile swelling that moving with respiration and from side to side. The provisional diagnosis was carcinoma of the stomach with metastasis to cervical lymph nodes or lymphoma, and a plan to investigate her abdominal mass was made.

On investigation, the hemoglobin was 90 g/l, with mild hypochromia on peripheral blood film examination. Other hematological findings, erythrocyte sedimentation rate, blood chemistry, electrocardiogram, X-ray (chest, abdomen standing) revealed no abnormality.

Bone marrow examination revealed mild iron-deficiency anemia. An upper gastrointestinal series showed a large space-occupying filling defect in the midcorpus, with thin lines of barium invaginating the mass (Fig. 1).

Ultrasonography showed a globular hyperechoic area inside the stomach with an acoustic shadowing (Fig. 2), but other viscera were normal. Fiberoptic endoscopy revealed a large, hard mass, black in color covered with hair extending from the corpus to the pylorus, partially occluding the lumen. It was difficult to biopsy and only two or three hairs were obtained on each attempt. The excision biopsy of the left cervical lymph nodes revealed caseous tubercular lymphadenitis. The patient underwent laparotomy and a large trichophytobezoar (Fig. 3), weighing 350 g was removed through gastrotomy. The patient had a satisfactory postoperative convalescence and was discharged 10 days later on a regimen of antitubercular treatment.

### DISCUSSION

The term "bezoar" refers to food or foreign matter that has undergone digestive changes in the gut of either animals or man. Most gastrointestinal bezoars result from inges-

Address for correspondence: Dr. G. M. Malik, M.D., F.A.C.G., R/O Kamerwari, SK Coloney, IA, Srinagar, 190 009, Kashmir, India.

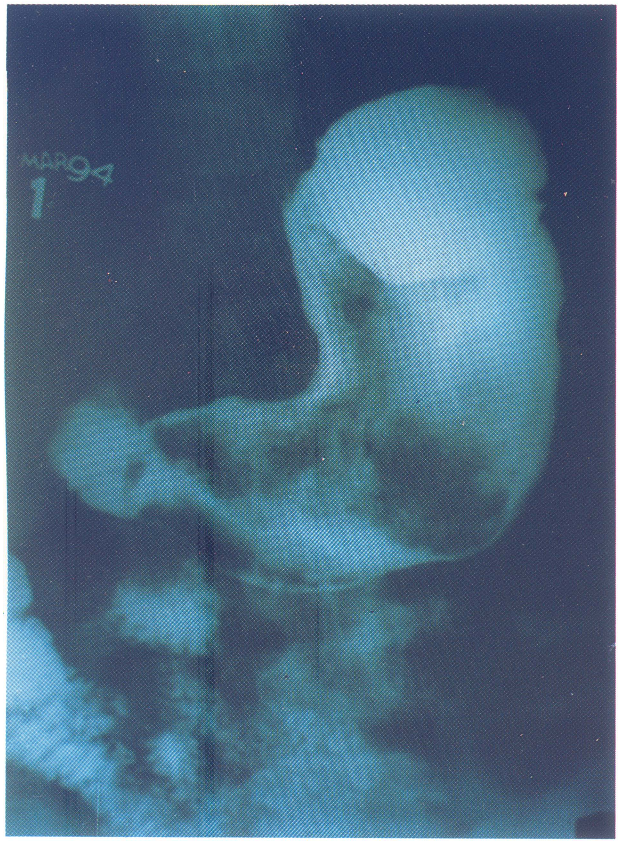

**Figure 1** Barium X-ray of the stomach showing a large filling defect in the midcorpus with thin lines of barium invaginating it and having no constant site of attachment, confirming the sonographic diagnosis.

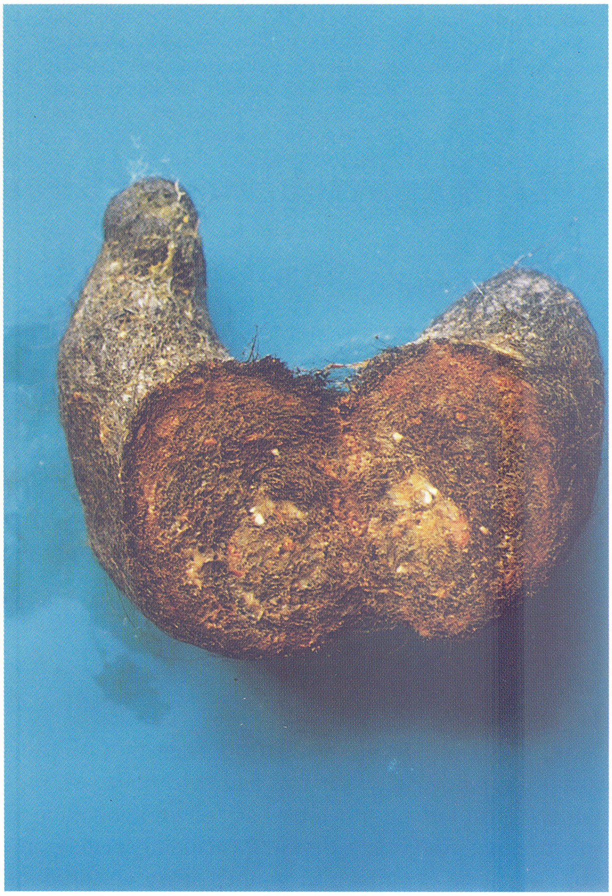

**Figure 3** Midtransverse section of a gastric trichophytobezoar.

tion of undigestible organic material, such as hair or fruit and vegetable fibers or both.[2,3] Some of the important predisposing factors include hypochlorhydria, diabetic gastroparesis, gastrectomy, vagotomy with drainage procedures, mental retardation and cimetidine therapy.[3–6] The symptoms include nausea, vomiting, abdominal pain, obstruction, and a mobile abdominal mass.[1,2,7,8] There have been case reports in which bezoars have been found as a complication of gastric carcinomas.[9]

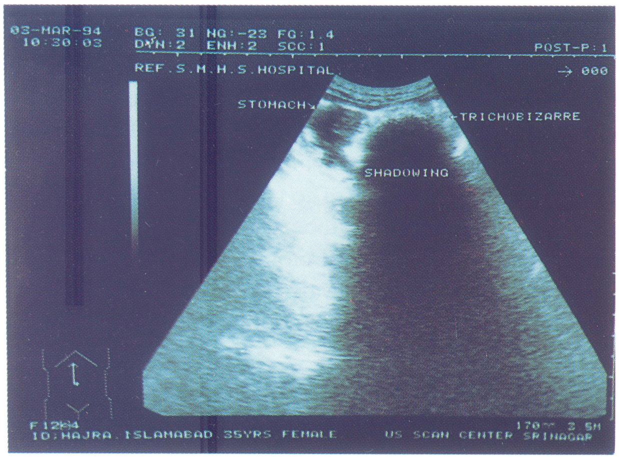

**Figure 2** Ultrasonography showing hyperechoic area inside the stomach with acoustic shadowing—a bezoar is suspected.

Ultrasonography and barium are of value in the diagnosis of trichophytobezoars. On ultrasonography, a gastric bezoar is viewed as a densely echogenic mass that casts a clean and complete acoustic shadow in the left upper quadrant. The clean shadow of a bezoar has been attributed to the densely packed food debris, bound together by the fine meshwork of hair and molded into a compact mass by gastric motility over a long period of time.

The classical appearance of a bezoar on a barium study is an intraluminal filling defect of variable size, with barium filling its interstices. Also, there is no constant site of attachment of a bezoar to the bowel wall, which rules out any intraluminal tumor.[10–13] The findings on ultrasonography and barium study in our patient conform with the above reports.

Our patient had an unusual presentation. She had no symptoms referable to the gastrointestinal system. The only findings she had were anemia, cervical lymphadenopathy, and a hard epigastric lump which suggested the provisional diagnosis of a gastric carcinoma or a lymphoma. Moreover, trichophytobezoar had not previously been reported in a patient with tuberculosis. Furthermore, trichophytobezoars weighing 350 g with no gastrointestinal symptom are unusual.

Small gastric phytobezoars may be removed endoscopically or may be treated with enzymes (pepsin, pancreatic enzyme concentrates, cellulase, or dehydrocholic acid), fragmentation by biopsy forceps, or possibly by medication (acetyl cystein or sodium bicarbonate).[1,8,14] Since the recurrence rate of trichophytobezoars is high, all such patients should be instructed to avoid consumption of raw fruits and stringy vegetables.[15]

### REFERENCES

1. Sharma V, Sahi RP, Misra NC, et al. Phytobezoar extending from stomach to cecum Indian Gastroenterol 1990;9:237.
2. Qureshi NH, Moris K, McDevit B. Trichobezoar—A condition to think of in case of mobile abd. mass. Ir Med J 1992;85:74.
3. Lal MM, Dhall JC. Trichobezoar; a collective analysis of 39 cases from India with a case report. Indian Pediatr 1975;12:351–353.
4. Raffin SB. Bezoars. In Sleisenger M, Fordtran J, eds. Gastrointestinal disease, 4th ed. Philadelphia: Saunders, 1988;741–745.
5. Brady P. Gastric phytobezoar consequent to delayed gastric emptying. Gastrointest Endosc 1978;24:159.
6. Nichols TW Jr. Phytobezoar formation: A new complication of cimetidine therapy. Ann Intern Med 1981;95:70.
7. Dasgupta H, Chandra S, Gupta M, et al. Trichobezoar—Clinical diagnosis. J Postgrad Med 1979;25:181.
8. Saxena C, Bhat N, Nagi B, et al. Bizarre trichobezoars. Indian Pediatr 1986;23:734–737.
9. Von Thiel D, De Belle R, Painter T, et al. Phytobezoar occurring as a complication of gastric carcinoma. Gastroenterology 1984;61:231.
10. William JW, Collins MC. A Chinese puzzle. Br J Radiol 1990;63:367–368.
11. Bidula MM, Rifkin MD, McCoy RI. Ultrasonography of gastric phytobezoar. J Clin Ultrasound 1986;14:49.
12. Verstanding AG, Klin B, Bloom RA, et al. Small bowel phytobezoars—Detection with radiography. Radiology 1989;172:705–707.
13. McCraken R, Jongiward R, Silver TM, et al. Gastric trichobezoar: Sonographic findings, Radiology 1986;161:123–124.
14. Harold M. Bruck MD, Ridgewood NJ. Gastric phytobezoar. JAMA 1975;231:26.
15. Norfleet R, Bickford R, Eckberg R. Gastric bezoars from swallowed string. JAMA 1978;240:855.

